# Distinctive CD56^dim^ NK subset profiles and increased NKG2D expression in blood NK cells of Parkinson’s disease patients

**DOI:** 10.1038/s41531-024-00652-y

**Published:** 2024-02-15

**Authors:** Stephen Weber, Kelly B. Menees, Jieun Park, Julian Agin-Liebes, Chih-Chun Lin, Roy N. Alcalay, Jae-Kyung Lee

**Affiliations:** 1 Department of Physiology and Pharmacology, University of Georgia College of Veterinary Medicine, Athens, GA USA; 2 Harrison College of Pharmacy, Auburn University, Auburn, AL USA; 3 Department of Neurology, Columbia University Medical Center, New York, NY USA; 4 Neurological Institute, Tel Aviv Sourasky Medical Center, Tel Aviv, Israel

**Keywords:** Neuroimmunology, Neuroimmunology

## Abstract

Mounting data suggest an important role for the immune system in Parkinson’s disease (PD). Previous evidence of increased natural killer (NK) cell populations in PD suggests a potential role of NK cells in the pathogenesis of the disease. Previous studies have analyzed NK cell populations using aggregation by variable expression of CD56 and CD16. It remains unknown what differences may exist between NK cell subpopulations when stratified using more nuanced classification. Here, we profile NK cell subpopulations and elucidate the expressions of activating, NKG2D, inhibitory, NKG2A, and homing, CX3CR1, receptors on NK cell subpopulations in PD and healthy controls (HC). We analyzed cryopreserved PMBC samples using a 10-color flow cytometry panel to evaluate NK cell subpopulations in 31 individuals with sporadic PD and 27 HC participants. Here we identified significant differences in the CD56^dim^ NK subset that changes with disease severity in PD. Furthermore, the expressions of NKG2D in all three NK cell subsets were significantly elevated in PD patients compared to HC. Notably, NKG2A expression in the CD56^bright^ NK subset increased in PD patients with longer disease duration but there were no changes in CX3CR1. In summary, our data suggests that changes in NK cells may be influenced by the clinical severity and duration of PD.

## Introduction

Recent evidence has implicated the role of neuroinflammation (thoroughly reviewed in^1^) in PD pathogenesis. Furthermore, studies have documented changes in the distributions of peripheral immune cells in PD patients^2,3^. Of particular interest are natural killer (NK) cells, an innate immune system population, traditionally associated with the destruction of malignant cells. This destruction occurs following signaling through activating and inhibitory receptors, resulting in cytotoxicity either through direct release of perforin and granzyme^4,5^ or through death receptor-mediated apoptotic signaling by TNF receptors, FasL and TNF-alpha-related apoptosis-inducing ligand (TRAIL) (reviewed in^6^). A recent study utilizing the preformed fibril (PFF) α-syn mouse model of PD demonstrated the infiltration of NK cells into the central nervous system (CNS) and observed alterations in their frequency and numbers in the periphery^7^.

Notably, in a preclinical mouse model of PD, systemic depletion of NK cells exacerbated synuclein pathology and motor symptoms, further implicating NK cells as a relevant cell type in the pathogenesis of PD^8^. Furthermore, this study revealed that NK cells internalized and degraded α-syn aggregates. Recent research in a murine model demonstrated a decline in NK cell numbers and showed functional deficits in α-syn clearance associated with age^9^. This highlights the necessity for further characterization of NK cells in PD patients to elucidate profile differences.

Human NK cells have been broadly classified based on the expression of cell surface markers, primarily cluster of differentiation (CD) 56 (neural cell adhesion molecule) and CD16 (Fcγ Receptor III), while being CD3 negative. These classifications have led to the identification of three primary NK cell subsets, characterized by variable expressions of CD56: (1) CD56^bright^, (2) CD56^dim^ and (3) CD56^−^. A recent study has distinguished additional distinct NK cell subpopulations based on variable expressions of both CD56 and CD16, defining six subpopulations: CD56^bright^CD16^−^, CD56^bright^CD16^dim^, CD56^dim^CD16^bright^, CD56^dim^CD16^−^, CD56^dim^CD16^dim^, and CD56^−^ CD16^+^ subpopulations^10^. The majority of circulating NK cells, upwards of 90%, fall into the CD56^dim^ NK subset, with the remaining 10% primarily consisting of CD56^bright^ NK subset and the smallest minority belonging to the CD56^−^ NK subset^10^. The developmental progression for NK cell expression of CD56 remains actively debated, but many consider the CD56^bright^ NK subset as the precursor to the CD56^dim^ NK subset^10^. The CD56^bright^ NK subset is considered to be the regulatory subset and serves an immunomodulatory role^11,12^. Moreover, abundant cytokines are produced by the CD56^bright^ subset including IFN-γ, TNF, IL-10, IL-13, and GM-CSF^10,11^. The CD56^dim^ subset is considered to be the more cytotoxic population and has increased levels of perforin, granzymes, and cytolytic granules^5,10,13^. It has also been demonstrated that the frequency of NK cell subsets changes with age. The CD56^bright^ subset has been shown to be decreased with age^14–16^ while the CD56^dim^ subset is increased^15^. The CD56^−^ NK cell subset is increased in chronic viral infections^17–19^ and display impaired cytotoxicity and cytokine production^18^.

In addition to expression of CD56 and CD16, differential expressions of activating and inhibitory receptors are shown to mediate NK cell activity^5,20,21^. The cumulative sum of activating and inhibitory signaling directly regulates NK cell effector functions.

Natural killer group 2D (NKG2D) receptor is an activating receptor constitutively expressed on NK cells^22^. Alterations in NKG2D have previously been reported in PD patients (summarized in^23^). The frequency of NKG2D + NK cells have been reported to be unchanged^24^ and increased in PD patient samples compared to healthy controls^25^. Natural killer group 2A (NKG2A) receptor is an inhibitory receptor expressed by NK cells that recognizes histocompatibility antigen, alpha chain E (HLA-E), also known as major histocompatibility complex (MHC) class I antigen E. NKG2A + NK cells have previously been reported to be decreased in PD patients compared to controls^24^. The interaction between chemokine C-X3-C motif receptor 1 (CX3CR1) and its ligand CX3CL1 (also known as fractalkine) mediates immune cell chemotaxis^26,27^. Expression of CX3CR1 has been shown to be essential for NK cell homing to the CNS and ameliorating disease in an experimental autoimmune encephalomyelitis (EAE) model of multiple sclerosis^28^. As NK cells have been demonstrated to be present in brains of patients with synucleinopathies and in mouse models of PD^7,8^, assessing CX3CR1 expression in PD patients warrants further investigation. In this study, we employed conventional flow cytometry to analyze changes in different subsets of NK cells in PD. Our study aimed to investigate the changes in NK cell subsets and receptor profiles that are associated with the clinical severity of PD and disease duration.

## Results

### Peripheral blood immune profile of idiopathic PD and Healthy Control

**Fig. 1:**
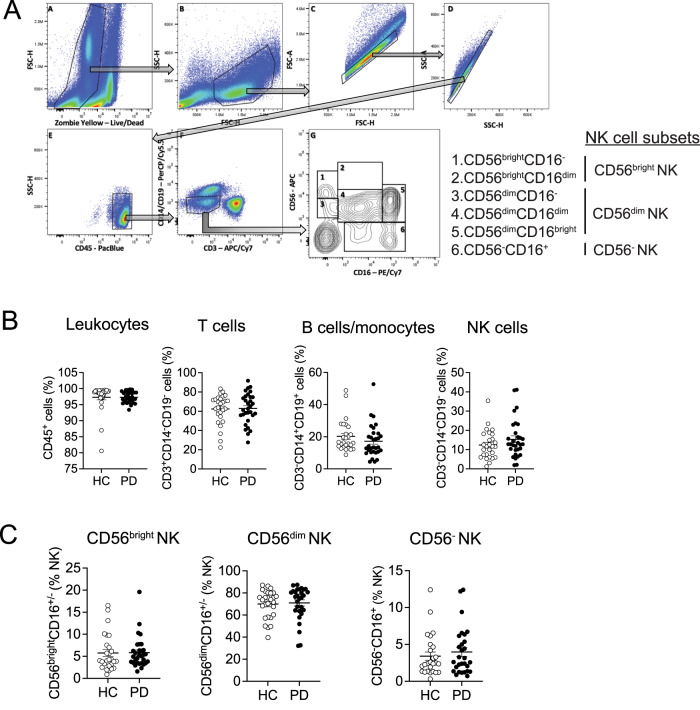
Leukocyte frequency is unchanged in PD patients compared to healthy controls. **A** NK cell gating strategy. Cells were first gated on forward scatter (FSC) and the live/dead marker Zombie Yellow to only include live cells. The live cell population was then gated on a FSC and side scatter (SSC). From the CD45+ population, NK cells were then gated from the CD14/19 negative CD3 negative population. NK subsets were gated based on CD56 and CD16 expression. **B** Frequency of total leukocytes, T cells, B cells/monocytes, and NK cells in PD patients and healthy controls. **C** Frequency of CD56^bright^, CD56^dim^ and CD56^−^ NK subsets in PD patients and healthy controls. Data were analyzed by Mann–Whitney test or Student *t*- test. Data represent mean ± SEM.

To validate the alteration of peripheral blood immune populations in PD, we utilized cryopreserved peripheral blood mononuclear cells (PBMCs) obtained from 58 donors, comprising 31 idiopathic PD patients and 27 healthy controls (HC), as outlined in Table 1. Flow cytometry was employed using our 10-parameter panel. The gating strategy involved the establishment of population gating using quantitatively determined antibody titrations. Gates were set based on single color and fluorescence minus one (FMO) control, with compensation to minimize fluorescent spill-over. To ensure accuracy, only live, single cell populations were analyzed to prevent non-specific binding artifacts or misrepresentation due to doublets, as described in the methods. The frequencies of cells expressing CD45^+^ (total leukocytes), CD3^+^CD14^−^CD19^−^ (T cells), CD3^−^CD14^+^CD19^+^ (B cells/monocytes), and CD3^−^CD14^−^CD19^−^ (NK cells) were found to be comparable between PD and control groups (Fig. 1B). To further validate the NK cell population, from CD3^−^CD14^−^CD19^−^ gating, we identified three NK subsets with six NK subpopulations: CD56^bright^ NK subset (CD56^bright^CD16^−^ and CD56^bright^CD16^dim^ NK subpopulations), CD56^dim^ NK subset (CD56^dim^CD16^bright^, CD56^dim^CD16^dim^, and CD56^dim^CD16^−^ NK subpopulations), and CD56^−^ NK subset (CD56^−^CD16^+^ NK subpopulations) as depicted in Fig. 1A. Our data showed that frequencies of CD56^bright^, CD56^dim^, and CD56^−^ NK subsets showed no significant differences between PD or HC (Fig. 1C).

**Table 1:** PD patient and healthy control demographics

Characteristics	PD		HC	
	Female, *N* = 17^a^	Male, *N* = 14^a^	Female, *N* = 15^a^	Male, *N* = 12^a^
Age	62.7 (±12.3)/66.6 (17.1)	64.1 (±10.7)/66.4 (14.3)	67.5 (±4.7)/68.2 (6.5)	67.2 (±11.4)/70.1 (10.3)
Disease duration, age, yr	6.3 (±4.5)/4.6 (4.9)	4.7 (±3.9)/3.0 (6.2)	0.0 (±0.0)/0.0 (0.0)	0.0 (±0.0)/0.0 (0.0)
Total UPDRS, score	28.4 (±16.6)/28.0 (19.3)	27.1 (±11.7)/25.0 (15.0)	–	–
Motor UPDRS score	19.1 (±11.6)/19.0 (11.8)	20.1 (±8.2)/18.5 (9.8)	–	–
LevEquiv	281.8 (±235.0)/300.0 (300.0)	261.8 (±245.1)/250.0 (400.0)	–	–
MoCA Total	26.9 (±2.3)/27.0 (4.0)	25.9 (±2.8)/27.0 (2.0)	27.4 (±1.8)/28.0 (1.8)	26.4 (±3.3)/27.0 (4.0)
*Group_disease duration*				
<4 yrs, *N* = 15	63.0 (±11.8)/66.6 (17.5)			
4+ yrs, *N* = 16	63.6 (±11.5)/66.1 (16.0)			
*Group_UPDRS socre*				
<20, *N* = 10	59.2 (±12.6)/61.3 (20.6)			
20+, *N* = 19	65.5 (±10.9)/68.9 (16.1)			

*PD* Parkinson’s disease, *UPDRS* unified Parkinson’s disease rating scale, *LevEquiv* levodopa equivalent daily dose, *MoCA* Montreal cognitive assessment, *SD* standard deviation.

^a^Mean (±SD)/median (IQR).

### Distinct CD56^dim^ NK cell profiles in PD are associated with disease severity

The discovery of distinct immune cell subsets or receptor profiles that serve as early diagnostic biomarkers presents an opportunity for earlier intervention and treatment. To investigate potential differences in the frequencies of NK cell subsets associated with disease severity, we divided PD samples into two groups based on the severity of their symptoms as measured by UPDRS scores. A total UPDRS score of <20 corresponded to mild symptoms (*n* = 10), while a total UPDRS scores of 20+ indicated moderate to severe symptoms (*n* = 19).

**Fig. 2:**
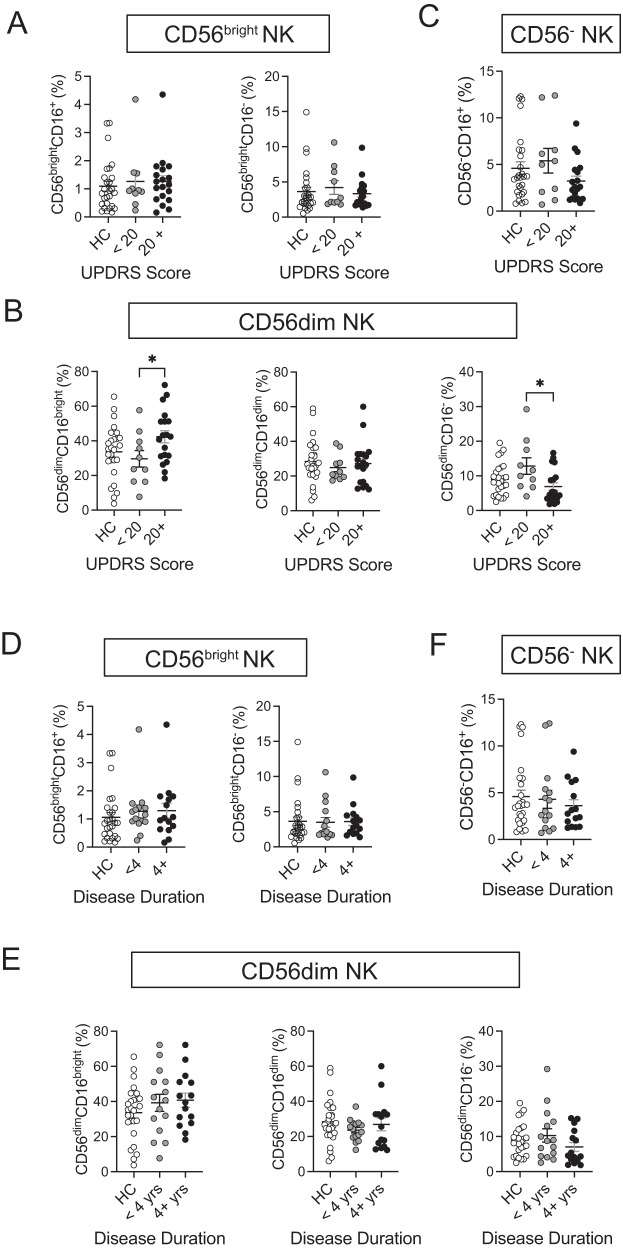
Distinct CD56^dim^ NK cell profiles in PD are associated with disease severity. Plots show frequencies of **A** CD56^bright^ NK subsets of total NK cells, **B** CD56^dim^ NK subset of total NK cells, and **C** CD56^−^ NK subset of total NK cells from HC and PD samples stratified by UPDRS score (<20, 20+). Plots show frequencies of **D** CD56^bright^ NK subsets of total NK cells, **E** CD56^dim^ NK subset of total NK cells, and **F** CD56^−^ NK subset of total NK cells from HC and PD samples stratified by disease durations (<4, 4+ years). Data represent mean ± SEM. Data were analyzed by one-way ANOVA or Kruskal-Willis test. Data represent mean ± SEM. *p < 0.05.

Within the CD56^bright^ NK subset, the frequency of the CD56^bright^CD16^+^ and CD56^bright^CD16^−^ NK subpopulation subsets did not show significant differences among individuals with mild PD (UPDRS score <20), those with PD (UPDRS score 20+), or HC (Fig. 2A). Within the CD56^dim^ NK subset, the frequency of the CD56^dim^CD16^bright^ subpopulation was increased in patients with higher UPDRS scores (20+) compared to patients with UPDRS score <20 (*p* = 0.0364), while no differences in the CD56^dim^CD16^dim^ NK subpopulation were observed (Fig. 2B). Conversely, CD56^dim^CD16^−^ NK cell frequencies decreased in patients with higher UPDRS score (20+) compared to in patients with mild PD (*p* = 0.0113) (Fig. 2B). The CD56^−^ NK subset did not any show significant differences among individuals with mild PD (UPDRS score <20), those with moderate/severe PD (UPDRS score 20+) or HC (Fig. 2C).

Next, we assessed the potential differences in the frequencies of three NK cell subsets between early and later stages of PD. We categorized PD samples into two groups based on their disease duration: those with a disease duration of less than 4 years as early-stage (*n* = 15), and those with 4 or more years as intermediate/late stages (*n* = 16). The frequencies of CD56^bright^, CD56^dim^, and CD56^−^ NK subsets did not show significant differences among individuals with early PD ( < 4 years), those with PD (4+ years) or HC (Fig. 2D–F).

### Altered frequencies and expressions of NKG2D in NK cell subsets are associated with disease severity and duration

The alterations in the activation status of NK cells during the progression of PD may provide insight into the potential mechanisms through which NK cells are involved in PD. NK cell activities are regulated by the collective balance of signals from activating and inhibitory receptors. Assessing the binary presence or absence, or frequency, of a receptor on NK cells may not provide the depth of detail necessary to understand changes at the receptor level. Therefore, to more accurately reflect the potential bias towards activation or inhibition, we also assessed the mean fluorescence intensity (MFI) to evaluate the variability in receptor expression intensity within NK cell profiles in addition to frequency.

**Fig. 3:**
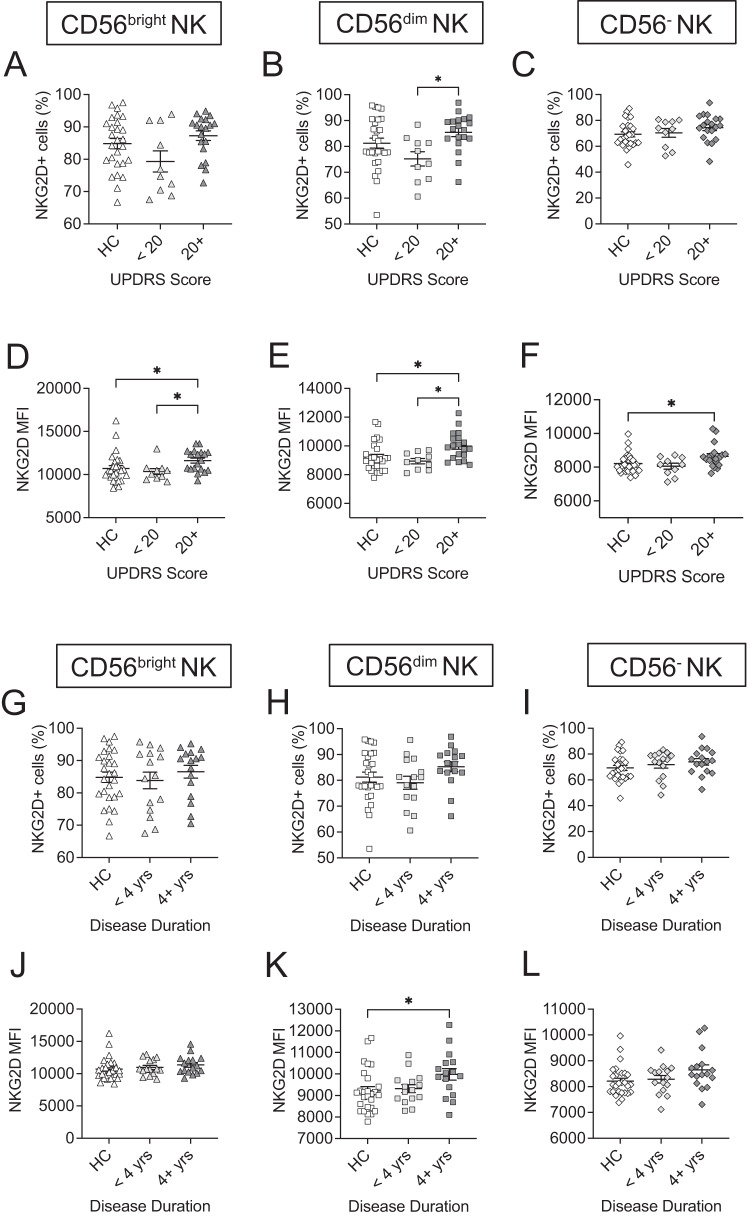
Altered frequencies and expressions of NKG2D in NK cell subsets are associated with disease severity and duration. Plots show **A** the frequency of NKG2D receptor expressing CD56^bright^ NK subset, **B** the frequency of NKG2D receptor expressing CD56^dim^ NK subset, and **C** the frequency of NKG2D receptor expressing CD56^−^ NK subset, grouped by UPDRS score (<20, 20+). Plots show MFIs of NKG2D+ CD56^bright^ NK subset (**D**), CD56^dim^ NK subset (**E**), and CD56^−^ NK subsets (**F**) grouped by UPDRS score (<20, 20+). Plots show **G** the frequency of NKG2D receptor expressing CD56^bright^ NK subset, **H** the frequency of NKG2D receptor expressing CD56^dim^ NK subset, and **I** the frequency of NKG2D receptor expressing CD56^−^ NK subset, grouped by disease duration (<4 years, 4+ years). Plots show MFIs of NKG2D+ CD56^bright^ NK subset (**J**), CD56^dim^ NK subset (**K**), and CD56^−^ NK subset (**L**) grouped by disease duration (<4 years, 4+ years). Data were analyzed One-way ANOVA or Kruskal–Willis test. Data represent mean ± SEM. *p < 0.05.

First, we evaluated changes in the frequency and expression level of NKG2D, an activating receptor, within NK cell populations in relation to UPDRS scores. Our assessment showed no significant differences in frequencies of NKG2D+ cells were observed in the CD56^bright^ NK subset (Fig. 3A). The frequency of NKG2D+ cells in the CD56^dim^ NK subset was significantly increased in individuals with UPDRS 20+ (*p* = 0.0180) compared to individuals with UPDRS scores <20 (Fig. 3B). No significant differences in frequencies of NKG2D+ cells were observed in the CD56^−^ NK subset (Fig. 3C).

To comprehensively understand receptor-level changes and accurately reflect potential biases toward activation or inhibition, we evaluate the expression intensity of NKG2D within the NK cell subsets. We observed significant increases in NKG2D expressions in the CD56^bright^ NK subsets of individuals with a UPDRS score 20+ compared to individuals with UPDRS score <20 (*p* = 0.0411) and HC (*p* = 0.0254) (Fig. 3D). Additionally, we found significant increases in NKG2D expressions in the CD56^dim^ NK subsets of individuals with a UPDRS score 20+ compared to individuals with UPDRS score <20 (*p* = 0.0263) and HC (*p* = 0.0207) (Fig. 3E). Furthermore, we observed a significant increase of NKG2D expression in the CD56^−^ NK subset of individuals with a UPDRS score 20+ compared to HC (*p* = 0.0449) (Fig. 3F). We also examined the correlation between the percent or MFI vs continuous total UPDRS score for the significant cases from ANOVA or Kruskal-Wallis (Table 2). Our data confirmed that the increased expressions of NKG2D in the CD56^bright^ (*r* = 0.197, *p* = 0.047), CD56^dim^ (*r* = 0.246, *p* = 0.013), and CD56^−^ (*r* = 0.229, *p* = 0.021) NK subsets were associated with higher UPDRS scores (Table 2).

**Table 2:** Correlation analyses with continuous total UPDRS score and disease duration for NKG2D

NK cell subset	Value	Correlation	p value	Score or Duration
CD56^dim^CD16^+/−^	%	0.124	0.212	UPDRS score
**CD56**^**bright**^**CD16**^**+/−**^	**MFI**	**0.197**	**0.047**	**UPDRS score**
**CD56**^**dim**^**CD16**^**+/−**^	**MFI**	**0.246**	**0.013**	**UPDRS score**
**CD56**^**−**^**CD16**^**+**^	**MFI**	**0.229**	**0.021**	**UPDRS score**
**CD56**^**dim**^**CD16**^**+/−**^	**MFI**	**0.210**	**0.020**	**Disease duration**

The *p*-value for the significance of the correlation coefficient is calculated under ${H}_{0}$ : the correlation coefficient is 0, that is, there is no linear relationship.

Next, we evaluated changes in the frequency of NKG2D within NK cell populations in relation to disease duration. Our data showed no significant differences in frequencies of NKG2D in the CD56^bright^ NK subset (Fig. 3G), CD56^dim^ NK subset (Fig. 3H) nor in the CD56^−^ NK subset among groups of HC, PD patients with disease duration <4 years or those with disease duration 4+ years (Fig. 3I). Importantly, the expression level of NKG2D expression were increased in individuals with a disease duration of 4+ years compared to HC in the CD56^dim^ subset (*p* = 0.0405) (Fig. 3K).

Our correlation test data confirmed that the increased expressions of NKG2D in the CD56^dim^ NK subsets (*r* = 0.210, *p* = 0.030) was associated with continuous disease duration (Table 2). However, the expression of NKG2D within the CD56^bright^ NK subset (Fig. 3J) and the CD56^−^ NK subset (Fig. 3L) was not significantly different among groups of HC, PD patients.

### Frequencies and expressions of NKG2A in NK cell subsets are altered in association with disease duration

**Fig. 4:**
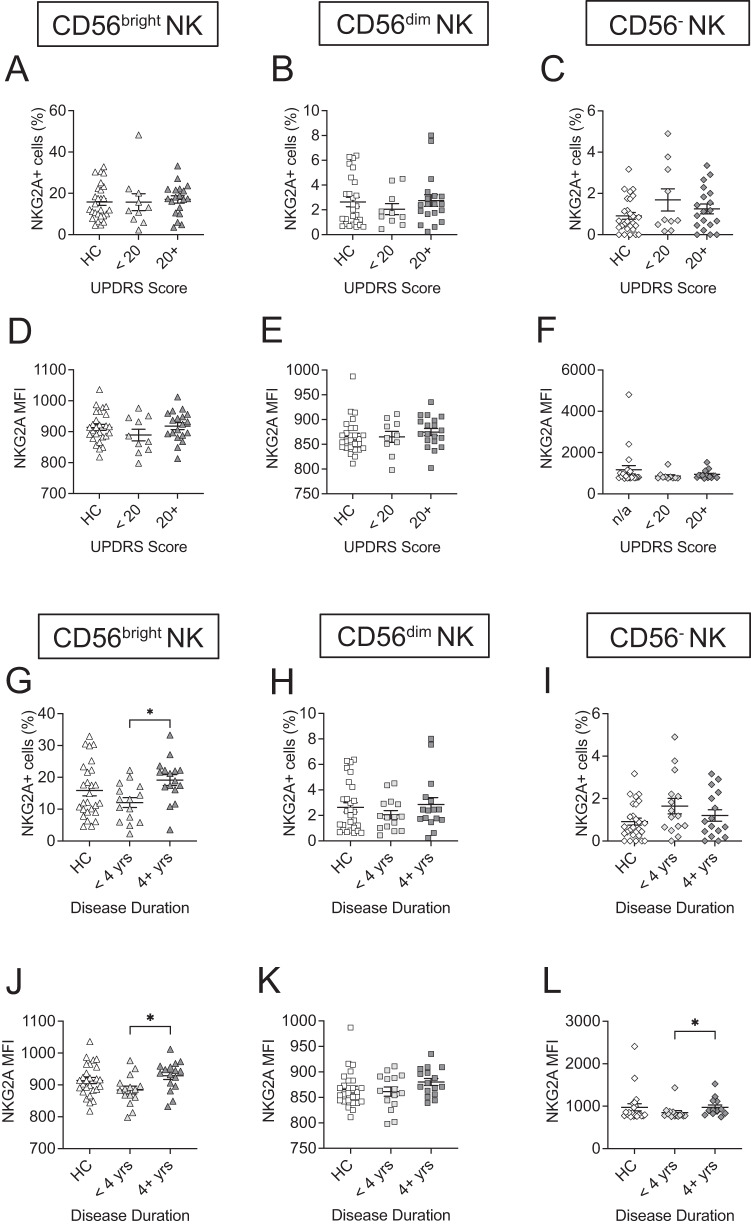
The frequencies and expressions of NKG2A in NK cell subsets are altered in association with disease duration. Plots show **A** the frequency of NKG2A receptor expressing CD56^bright^ NK subset, **B** the frequency of NKG2A receptor expressing CD56^dim^ NK subset, and **C** the frequency of NKG2A receptor expressing CD56^−^ NK subset, grouped by UPDRS score (<20, 20+). Plots show MFIs of NKG2A+ CD56^bright^ NK subset (**D**), CD56^dim^ NK subset (**E**), and CD56^−^ NK subsets (**F**) grouped by UPDRS score (<20, 20+). Plots show **G** the frequency of NKG2A receptor expressing CD56^bright^ NK subset, **H** the frequency of NKG2A receptor expressing CD56^dim^ NK subset, and **I** the frequency of NKG2A receptor expressing CD56^−^ NK subset, grouped by disease duration (<4 years, 4+ years). Plots show MFIs of NKG2A+ CD56^bright^ NK subset (**J**), CD56^dim^ NK subset (**K**), and CD56^−^ NK subset (**L**) grouped by disease duration (<4 years, 4+ years). Data were analyzed one-way ANOVA or Kruskal-Willis test. Data represent mean ± SEM. *p < 0.05.

Alterations in inhibitory receptor NKG2A represent an alternative mechanism of change in NK cell function. We evaluated changes in the frequency of NKG2A within NK cell populations in relation to disease severities. Analyses of the frequencies of NKG2A expressing cells showed no significant differences in the CD56^bright^ NK subset (Fig. 4A), CD56^dim^ NK subset (Fig. 4B) nor in the CD56^−^ NK subset among groups of HC, PD patients with UPDRS scores <20, or those with scores 20+ (Fig. 4C). Similarly, no significant differences in the expressions of NKG2A within NK cell subsets in relation to UPDRS scores (Fig. 4D), CD56^dim^ NK subset (Fig. 4E) nor in the CD56^−^ NK subset (Fig. 4F) were observed.

Next, we evaluated changes in the frequencies of NKG2A within NK cell populations in relation to disease duration. We observed a significant increase in the frequency of NKG2A-expressing CD56^bright^ NK subset in individuals with a disease duration of 4+ years compared to those with a disease duration of <4 years (*p* = 0.0371) (Fig. 4G). Our data showed no significant differences in frequencies of NKG2A in the CD56^dim^ NK subset (Fig. 4H) or the CD56^−^ NK subset (Fig. 4I) among groups of HC, PD patients with early stages (<4 years), or those with later stages (4+ years).

Importantly, the expression levels of NKG2A were increased in individuals with a disease duration of 4+ years compared to those with disease duration <4 years in the CD56^bright^ (*p* = 0.0390) (Fig. 4J) and the CD56^−^ NK subset (*p* = 0.0409) (Fig. 4L). However, the expression of NKG2A within the CD56^dim^ NK subset did not significantly differ among the three groups (Fig. 4K). We examined the correlation between the percent or MFI and continuous disease duration for the significant cases identified from ANOVA or Kruskal-Wallis. Our data indicates that increased expressions of NKG2A in the CD56^−^ NK subsets were correlated with higher disease duration (*r* = 0.221, *p* = 0.033) (Table 3).

**Table 3:** Correlation analyses with disease duration for NKG2A

NK cell subset	Value	Correlation	*p* Value	Score or duration
CD56^bright^CD16^+/^^−^	%	0.137	0.154	Disease duration
CD56^bright^CD16^+/^^−^	MFI	0.071	0.465	Disease duration
**CD56**^**−**^**CD16**^**+**^	**MFI**	**0.221**	**0.033**	**Disease duration**

The p-value for the significance of the correlation coefficient is calculated under ${H}_{0}$ : the correlation coefficient is 0, that is, there is no linear relationship.

### Frequencies and expressions of CX3CR1 in NK cell subsets are not changed with disease severity and duration

Alterations in activation and inhibitory receptor profiles on NK cells may not account for all contributing factors in PD progression. The specific tissue localization of NK cells is regulated by chemokine-induced migration from the blood, with CX3CR1 playing a crucial role in NK cell infiltration into the CNS. To understand this, we assessed the status of the NK homing receptor, CX3CR1, within NK cell populations in relation to disease severity and duration. Our results showed more than 90% of the CD56^dim^ and CD56^−^ NK subsets express CX3CR1 receptor (Supplementary Fig. 1B, C). We found no significant differences in the frequencies of CX3CR1 expressing NK cell subsets among HC and PD patients with different disease severity (Supplementary Fig. 1A–C). We found no significant differences in the expression of CX3CR1 among HC compared to PD groups (Supplementary Fig. 1D–F).

Similarly, when we compared the frequencies and expression of CX3CR1 among the group of HC and PD with different disease duration, there were no significant differences in frequencies (Supplementary Fig. 1G–I) or CX3CR1 expression across NK cell subsets (Supplementary Fig. 1J–L).

## Discussion

Our findings indicate that there is no notable disparity in the frequency of NK cells between HC and PD patients when employing the CD3^−^CD14^−^CD19^−^ gating strategy (Fig. 1). This result is not surprising as the gating strategy differs from previously published approaches which have primarily focused on analyzing NK cell subsets using varying levels of CD56 and CD16 expression^24,25^. In addition to CD56^+^ and CD16^+^, our study includes CD56^−^ and CD16^−^ populations that have only recently gained attention, all within the CD3^−^CD14^−^CD19^−^ gating strategy. By defining these profiles, we can gain insights into potential biomarkers that classify critical intervention windows. In this assessment, we also analyzed receptor expression profiles within NK cell subsets, including the variable expressions of NKG2D, NKG2A, and CX3CR1 on CD56^bright^, CD56^dim^, and CD56^−^ NK subsets which delineated a profile for the subtle changes within these NK cell populations in PD patients.

The CD56^dim^ NK subset represents the major subset accounting more than 90% of NK cells in the blood with primarily cytotoxic functions. Although functional differences of each NK subpopulation within the NK cell subsets remain unclear, a recent study suggested that within the CD56^dim^ NK subset, CD56^dim^CD16^bright^ NK subpopulation losses the expression of CD16 upon activation which transforms them to CD56^dim^CD16^−^ NK cells which include the highest cytotoxic properties^29^.

CD56^dim^CD16^dim^ NK cells are more degranulated than CD56^dim^CD16^bright^ NK cells and associated with HIV-1 infected individuals^20^. Our data showed that the percentage of CD56^dim^CD16^bright^ NK subpopulation significantly increased while that of CD56^dim^CD16^−^ NK subpopulation was decreased in patients with higher UPDRS scores (Fig. 2).

The CD56^bright^ NK subset represents a smaller population, excels in cytokine production like IFN-γ, TNF or IL-10, and are found more abundantly in secondary lymphoid organs, peripheral tissues, or area of inflammation^30,31^. In the central nervous system (CNS), the majority of NK cells in human cerebrospinal fluid (CSF) are CD56^bright^ NK subset^32,33^. These CD56^bright^ NK cells are found in lymph, where lymphatic vessels associated with the CNS^34^ suggesting they may have a high migratory capacity^35^. In mice, NK cells dampen CNS inflammation by eliminating autoimmunogenic T cells and microglia^36,37^ and are involved in cytotoxicity of immature or damaged neurons^38,39^ suggesting functional significance of NK cells as innate immune cells in CNS disorders.

In our assessment, we focused on NKG2D and NKG2A to understand the involvement of activating and inhibitory signaling, respectively, in immune context changes that inform PD progression and severity. Our analysis reveals significant increase in the expression of NKG2D in the CD56^bright^ and CD56^dim^ NK subsets with higher UPDRS scores in PD patients compare to HC (Fig. 3), providing valuable insights into the immunomodulatory and cytotoxicity contexts that NK cells may mediate in PD patients. We demonstrate that increased NKG2D expressions in all three CD56^bright^, CD56^dim^, and CD56^−^ NK subsets were weakly but significantly associated with higher UPDRS scores in PD patients (Table 2), suggesting that a potential link between NKG2D expression and the severity of Parkinson’s symptoms, as measured by the UPDRS scores. Interestingly, we also observed a significant increase in NKG2D expression in CD56^dim^, and CD56^−^ NK subsets with increasing disease duration compared to HC. These may imply an increased NK cells activation of may correlate with changes in immune activity that underlie variations in disease severity.

Interestingly, our results reveal a significant increase in the frequency and expression of NKG2A in the CD56^bright^ NK subset in patients with a PD duration of 4+ years (Fig. 4). This suggests a potential disproportionate bias in activation signaling via NKG2D in the CD56^bright^ NK subset in patients with PD patients, which may contribute to pathological changes. Further investigation is needed to define the role of these changes in PD.

The specific localization of NK cells in various tissues is regulated by chemokine- induced migration from the peripheral blood, with CX3CR1 expression being crucial for NK cell infiltration into the CNS^28,40,41^. Notably, the human CD56^dim^ NK subset expresses high levels of CX3CR1, making them selectively responsive to CX3CL1^42^.

Our data supports this observation, indicating that more than 90% of the CD56^dim^ NK subset expresses CX3CR1. Interestingly, we found no significant changes in CX3CR1 expression within NK cell subsets concerning PD severity or duration when compared to HC (Supplementary Fig. 1). This suggests that the migration and infiltration capacities of NK cells may remain relatively unchanged in PD.

The correlation analyses concerning the daily dose of levodopa did not yield significant correlations with the changed we observed. In this study, PD patient’s cohort is in relatively mild or moderate stages, as indicated in Table 1. We clarify that our cohort was primarily designed for the purpose of comparing PD patients to control groups, rather than being tailored for epidemiological study. Our primary objective was to intricately characterize NK cells with the aim of identifying subtle distinctions among NK cell subsets. These distinctions are crucial for recognizing significant differences in PD progression. While we recognize the necessity of further validation in carefully phenotype cohorts to gain more comprehensive understanding of NK cell alteration in PD, our data highlight significant changes in the percentage of CD56^dim^ NK subset within PD subgroups. Although there were no significant changes in the percentage of the CD56^dim^ NK subset between PD patients and HC, the significant changes we identified within the PD subgroups are still of great interest and significance. This data underscores how NK cell subsets change throughout the course of disease and with disease severity. Importantly, our data demonstrates a significant increase in NKG2D MFI in the CD56^dim^ NK subset in PD patients with UPDRS scores of 20 or higher and disease duration of 4 years or more compared to HC. Furthermore, our analyses reveal an increase in the levels of NKG2D in NK cells across PD stages and correlation analyses.

Our study established a standard NK profiling approach. Future research should strive to aim further determine the changes in the frequency and expression of distinct repertoires of activating and inhibitory receptors, as well as cytokine/chemokine receptors in various NK subsets. This research will contribute to a deeper understanding of their functions or migratory behaviors in PD. Additionally, investigating how NK cells interact with neighboring cell populations can provide insights into their role in shaping the immune environment which the CNS or periphery including gut.

Defining this relationship lays the groupwork for the development of targeted interventions aimed at altering the immune context that contributes to the progression of PD.

## Methods

### Participants and PBMC samples

Blood samples were obtained from the LRRK2 Biobanking Initiative site at the Columbia University Medical Center under the direction of Dr. Roy Alcalay (Columbia IRB approved Protocol #AAAP9604). The LRRK2 Biobanking Initiative is coordinated and funded by The Michael J. Fox Foundation for Parkinson’s Research. All clinical study procedures were approved by the Columbia University Institutional Review Board, and all participants signed informed contents. The current analysis excluded participants with systemic inflammation or past history of metastatic cancers or autoimmune diseases, resulting in a cohort of 31 idiopathic PD patients (17 females and 14 males) and 27 control subjects (15 females and 12 males). Summary of sample demographics are outlined in Table 1. To investigate potential differences in the frequencies of NK cell subsets associated with disease severity, we divided PD samples into two groups based on the severity of their symptoms as measured by UPDRS scores: A total UPDRS score of <20 corresponded to mild symptoms (*n* = 10), while a total UPDRS scores of 20+ indicated moderate to severe symptoms (*n* = 19). Analyses excluded two participants with missing UPRRS scores. To investigate potential differences in the frequencies of NK cell subsets associated with disease stages, we divided PD samples into two groups based on disease duration: those with a disease duration of less than 4 years as early-stage (*n* = 15), and those with 4 or more years as intermediate/late stages (*n* = 16).

### Preparation of PBMCs

PBMCs from blood samples were collected in sodium citrate-coated tubes following ficoll gradient separation, suspended in dimethyl sulfoxide (DMSO) cryopreservation buffer, and stored at −80 °C by the Dr. Roy Alcalay Lab at Columbia University. Samples were delivered on dry ice and upon delivery samples were immediately stored at −80 °C until use. PBMC processing was adapted from Barcelo et al.^43^. Cryopreserved samples were submerged halfway for 60 s in a 37 °C water bath. Immediately prior to full sample thaw 1 mL of pre-warmed (37 °C) complete RPMI (RPMI, 10% FBS, 1% Pen/Strep) was added dropwise, pipetted against the tube wall. Thawed PBMCs were poured into a 15 mL conical tube containing 5 mL of pre- warmed (37 °C) complete RMPI. Cryovials were rinsed with 2 mL of pre-warmed (37 °C) complete RPMI and then poured into the previously used conical tube with cell mixture. PBMCs were incubated for 5 minutes in a 37 °C water bath. PBMCs were then pelleted for 10 min at 1500 rpm, at room temperature. Supernatant was discarded and 1 mL of pre-warmed (37 °C) complete RPMI with 50 U/mL of DNase (Roche, Cat# 04-716- 728-001, 10 units/µL) was added, resuspension was done without pipetting. PBMCs were then incubated for 1 h at 37 °C in a water jacketed incubator (5% CO_2_, 95% humidity) with tube cap loosened. Following incubation PBMCs were pelleted and resuspended for counting in preparation for flow cytometry.

### Flow cytometry

All antibodies and Live/Dead stain: CD45-PacBlue (1:200, clone HI30, BioLegend), CD14-PerCP/Cy5.5 (1:100, HCD14, BioLegend), CD19-PerCP/Cy5.5 (1:100, HIB19, BioLegend), CD3-APC/Cy7 (1:50, HIT3a, BioLegend), CD56-APC (1:100, HCD56, BioLegend), CD16-PE/Cy7 (1:100, 3G8, BioLegend), NKG2D-FITC (1:100, 1D11, BioLegend), NKG2A-PE (1:100, 131411, R&D), CX3CR1-BV711 (1:100, 2A9-1, BioLegend), and Zombie Yellow (423103, BioLegend) were individually titrated using Veri-Cells (Cat# 425001, BioLegend) to determine optimal staining concentrations.

Prepared PBMCs were transferred to a 96-well plate, pelleted (1500 rpm for 5 min at room temp) and washed with PBS. PBMCs were incubated with Zombie Yellow (1:500) for 20 min, at room temperature, in the dark. PBMCs were washed with FACS Buffer (0.1% BSA, 1 mM EDTA, 0.01% Sodium Azide, and PBS) and pelleted. Samples were then resuspended in FACS Buffer with Human TruStain FcX (1:20, Cat# 422302, BioLegend) for 10 minutes at room temperature. Samples were then pelleted and resuspended in antibody master mix at a ratio of 1 × 10^6^ cells/100 µL master mix. Cells were incubated for 20 min at room temperature in the dark. Cells pelleted and washed twice with PBS. Prior to analysis samples were pelleted and resuspended in 2% paraformaldehyde. Samples were analyzed immediately after preparation. Single-color controls were prepared for each run using UltraComp eBeads (Cat# 01-2222-42, Thermo Fisher Scientific).

For flow cytometry acquisition, samples were analyzed using a NovoCyte Quanteon Flow Cytometer (Agilent Technologies). The system used has 4 excitation lasers: 405 nm, 488 nm, 561 nm and 640 nm. Prior to sample analysis, instrumentation performance was evaluated by the quality control (QC) procedure, experiments were only run if performance was optimal. To establish study standardization longitudinally, antibody titration was performed, and optimal antibody concentrations were determined. Using optimal antibody concentrations, instrument gain settings were established for each parameter by evaluation of single-positive signals and confirmation that all positive events were below the maximum dynamic range of the instrument (7.2 log). Using established gains for all parameters, compensation was calculated, fluorescence minus one (FMO) controls, and single color controls were collected, to ensure downstream analysis accuracy of determined positive event populations. A minimum of 100,000 events were collected per sample to ensure robust breadth of population distributions.

Analysis of flow cytometry data was carried out using FlowJo 10.8 (BD Biosciences).

### Statistical analyses

The normal distribution of the values was assessed using the histograms, density plots, normal QQ-plots, and Shapiro-Wilk test. We used the two-sample Student *t*-test for normally distributed data and the Mann-Whitney *U* test for non-normally distributed data to compare two groups. When comparing more than two groups, We employed one-way analysis of variance (ANOVA) with post hoc two-sample *t*-test for normally distributed data. Bonferroni correction was applied to adjust for multiple comparison. For non-normally distributed data, Kruskal-Wallis was employed followed by Dunn’s test for pairwise comparisons. For the correlation test, we used the Kendall correlation test. The data were presented as the mean with standard deviation (SD) or standard error of the mean (SEM), as indicated figure legends. A *p*-value of less than 0.05 was considered statistically significant. For statistical analysis, we used Graphpad Prism (Graphpad Software, v10.0.3) and R.

### Reporting summary

Further information on research design is available in the Nature Research Reporting Summary linked to this article.

### Supplementary information

Supplementary File

Reporting summary

## Data Availability

The data that support the findings of this study are available from the corresponding author upon request.
