# Supplementary material for: Distinctive CD56dim NK subset profiles and increased NKG2D expression in blood NK cells of Parkinson’s disease patients
Source: NPJ Parkinsons Dis. 2024 Feb 15;10:36. doi: 10.1038/s41531-024-00652-y (PMC10869354; doi:10.1038/s41531-024-00652-y)
Supplement: Supplementary file 1 — Supplementary File [file 41531_2024_652_MOESM1_ESM.pdf]

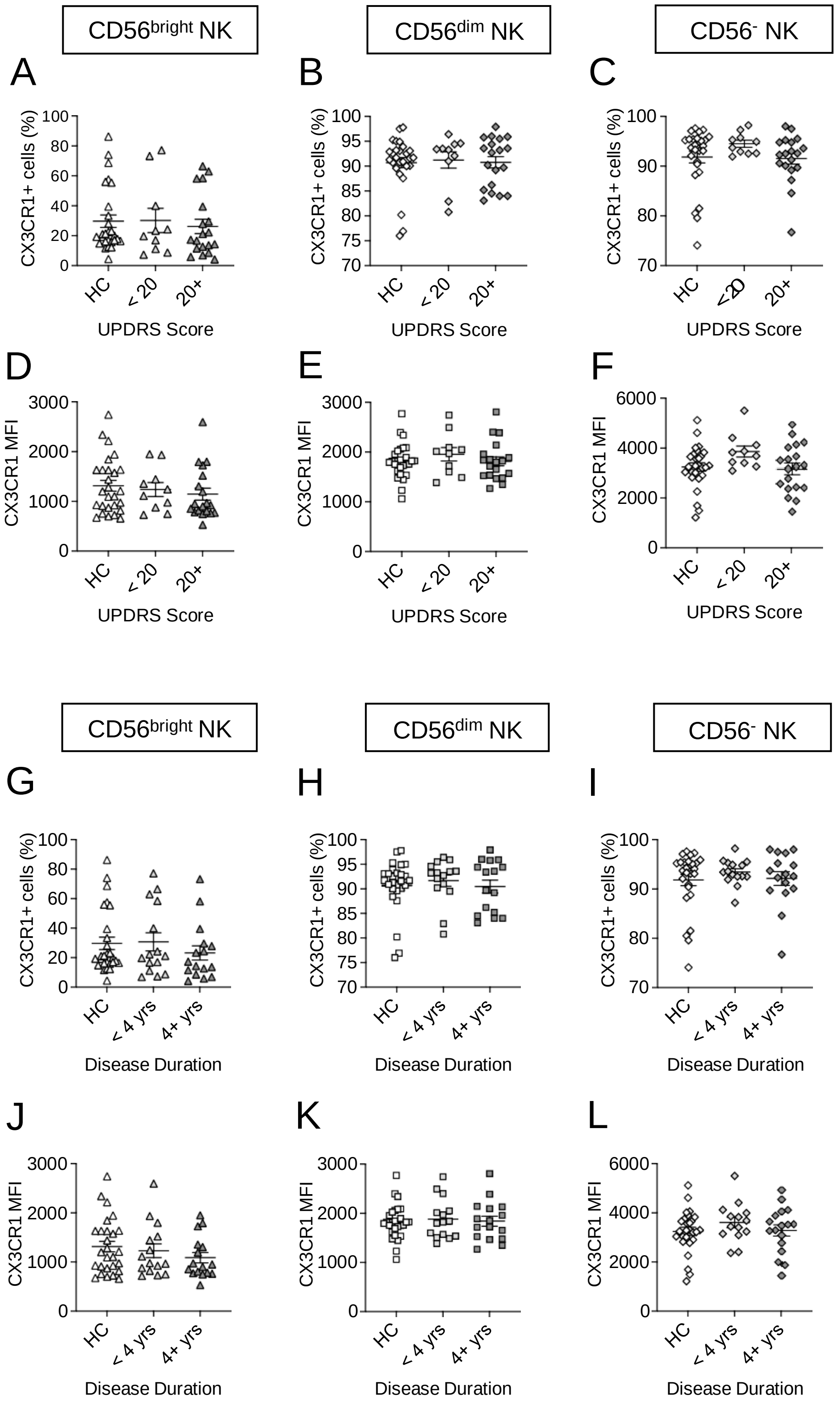

CD56bright NK
CD56dim NK
CD56- NK
A
B
C
CX3CR1+ cells (%)
HC
< 20
20+
UPDRS Score
D
E
F
CX3CR1 MFI
G
H
I
< 4 yrs
4+ yrs
Disease Duration
J
K
L

Supplementary Figure 1. Frequencies and expressions of CX3CR1 in NK cell subsets are not changed with disease severity and duration. Plots show **(A)** the frequency of CX3CR1 receptor expressing $CD56^{bright}$ NK subset, **(B)** the frequency of CX3CR1 receptor expressing $CD56^{dim}$ NK subset, and **(C)** the frequency of CX3CR1 receptor expressing $CD56^{-}$ NK subset, grouped by UPDRS score (<20, 20+). Plots show MFIs of CX3CR1+ $CD56^{bright}$ NK subset **(D)**, $CD56^{dim}$ NK subset **(E)**, and $CD56^{-}$ NK subsets **(F)** grouped by UPDRS score (<20, 20+). Plots show **(G)** the frequency of CX3CR1 receptor expressing $CD56^{bright}$ NK subset, **(H)** the frequency of CX3CR1 receptor expressing $CD56^{dim}$ NK subset, and **(I)** the frequency of CX3CR1 receptor expressing $CD56^{-}$ NK subset, grouped by disease duration (<4 years, 4+ years). Plots show MFIs of CX3CR1+ $CD56^{bright}$ NK subset **(J)**, $CD56^{dim}$ NK subset **(K)**, and $CD56^{-}$ NK subset **(L)** grouped by disease duration (<4 years, 4+ years). Data were analyzed One-way ANOVA or Kruskal-Willis test. Data represent mean ± SEM.
