# Supplementary material for: Distinctive CD56dim NK subset profiles and increased NKG2D expression in blood NK cells of Parkinson’s disease patients
Source: NPJ Parkinsons Dis. 2024 Feb 15;10:36. doi: 10.1038/s41531-024-00652-y (PMC10869354; doi:10.1038/s41531-024-00652-y)
Supplement: Supplementary file 2 — Reporting summary [file 41531_2024_652_MOESM2_ESM.pdf]

nature portfolio

Corresponding author(s): Jae-Kyung Lee

Last updated by author(s): Oct 17, 2023

# Reporting Summary

Nature Portfolio wishes to improve the reproducibility of the work that we publish. This form provides structure for consistency and transparency in reporting. For further information on Nature Portfolio policies, see our Editorial Policies and the Editorial Policy Checklist.

## Statistics

For all statistical analyses, confirm that the following items are present in the figure legend, table legend, main text, or Methods section.

| n/a | Confirmed | |
|---|---|---|
| ☐ | ☒ | The exact sample size (*n*) for each experimental group/condition, given as a discrete number and unit of measurement |
| ☐ | ☒ | A statement on whether measurements were taken from distinct samples or whether the same sample was measured repeatedly |
| ☐ | ☒ | The statistical test(s) used AND whether they are one- or two-sided<br>*Only common tests should be described solely by name; describe more complex techniques in the Methods section.* |
| ☐ | ☒ | A description of all covariates tested |
| ☐ | ☒ | A description of any assumptions or corrections, such as tests of normality and adjustment for multiple comparisons |
| ☐ | ☒ | A full description of the statistical parameters including central tendency (e.g. means) or other basic estimates (e.g. regression coefficient) AND variation (e.g. standard deviation) or associated estimates of uncertainty (e.g. confidence intervals) |
| ☐ | ☒ | For null hypothesis testing, the test statistic (e.g. *F*, *t*, *r*) with confidence intervals, effect sizes, degrees of freedom and *P* value noted<br>*Give P values as exact values whenever suitable.* |
| ☒ | ☐ | For Bayesian analysis, information on the choice of priors and Markov chain Monte Carlo settings |
| ☒ | ☐ | For hierarchical and complex designs, identification of the appropriate level for tests and full reporting of outcomes |
| ☐ | ☒ | Estimates of effect sizes (e.g. Cohen's *d*, Pearson's *r*), indicating how they were calculated |

*Our web collection on statistics for biologists contains articles on many of the points above.*

## Software and code

Policy information about availability of computer code

| | |
|---|---|
| Data collection | No software/code was used to collect the data in this study. |
| Data analysis | For flow data anlaysis, we used FlowJo 10.8 (BD Biosciences). For statistical analysis, we used Graphpad Prism (Graphpad Software, v10.0.3) and R. |

For manuscripts utilizing custom algorithms or software that are central to the research but not yet described in published literature, software must be made available to editors and reviewers. We strongly encourage code deposition in a community repository (e.g. GitHub). See the Nature Portfolio guidelines for submitting code & software for further information.

## Data

Policy information about availability of data

All manuscripts must include a data availability statement. This statement should provide the following information, where applicable:

- Accession codes, unique identifiers, or web links for publicly available datasets
- A description of any restrictions on data availability
- For clinical datasets or third party data, please ensure that the statement adheres to our policy

The data that support the findings of this study are available from the corresponding author upon request.

## Research involving human participants, their data, or biological material

Policy information about studies with human participants or human data. See also policy information about sex, gender (identity/presentation), and sexual orientation and race, ethnicity and racism.

| | |
|---|---|
| Reporting on sex and gender | Thirty-one idiopathic PD patients (17 female and 14 males) and 27 control subject (15 females and 12 males). |
| Reporting on race, ethnicity, or other socially relevant groupings | The study in this manuscript did not specify race, ethnicity, or other socially relevant groups. |
| Population characteristics | Population characteristics are presented in Table 1. |
| Recruitment | Participants were recruited by the LRRK2 Biobanking Initiative site at the Columbia University Medical Center. PMBC samples from the participants with no metastatic cancers or autoimmune diseases or the presence of accompanying inflammatory conditions were included in this study. |
| Ethics oversight | The study was approved by the institutional review board at the Columbia University Medical Center. |

Note that full information on the approval of the study protocol must also be provided in the manuscript.

# Field-specific reporting

Please select the one below that is the best fit for your research. If you are not sure, read the appropriate sections before making your selection.

☒ Life sciences ☐ Behavioural & social sciences ☐ Ecological, evolutionary & environmental sciences

For a reference copy of the document with all sections, see nature.com/documents/nr-reporting-summary-flat.pdf

# Life sciences study design

All studies must disclose on these points even when the disclosure is negative.

| | |
|---|---|
| Sample size | The sample size used in this study was based on previous published reports in the field as an acceptable sample size for a cross-sectional anlaysis between PD and age-matched control. Thirty-one idiopathic PD patients and 27 control subject. Summary of sample demographics and statistics are outlined in the manuscript Table 1. |
| Data exclusions | As described in the 'Participants and PMBC samples' paragraph of the Materials and Methods section, two PD participants with missing UPDRS scores were excluded from the analyses in disease severity but included in the analyses of disease duration. |
| Replication | All data from the control and PD population were consistently replicated in each experiment. We encountered no issue with reproducibility and are confident in the validity of our findings. |
| Randomization | Patients from the PD population were recruited randomly with inclusion criteria of confirmed disease according to the Movement Disorder Society clinical diagnostic criteria for PD, age appropriate for disease. Controls were recruited as age-matched. |
| Blinding | All samples were number coded. Patients groupings were blinded during processing PBMC and flow cytometry experiments. |

# Reporting for specific materials, systems and methods

We require information from authors about some types of materials, experimental systems and methods used in many studies. Here, indicate whether each material, system or method listed is relevant to your study. If you are not sure if a list item applies to your research, read the appropriate section before selecting a response.

## Materials & experimental systems

| n/a | Involved in the study | |
|---|---|---|
| ☐ | ☒ | Antibodies |
| ☒ | ☐ | Eukaryotic cell lines |
| ☒ | ☐ | Palaeontology and archaeology |
| ☒ | ☐ | Animals and other organisms |
| ☒ | ☐ | Clinical data |
| ☒ | ☐ | Dual use research of concern |
| ☒ | ☐ | Plants |

## Methods

| n/a | Involved in the study | |
|---|---|---|
| ☒ | ☐ | ChIP-seq |
| ☐ | ☒ | Flow cytometry |
| ☒ | ☐ | MRI-based neuroimaging |

# Antibodies

Antibodies used

CD45-PacBlue (1:200, clone HI30, BioLegend), CD14-PerCP/Cy5.5 (1:100, HCD14, BioLegend), CD19-PerCP/Cy5.5 (1:100, HIB19, BioLegend), CD3-APC/Cy7 (1:50, HIT3a, BioLegend), CD56-APC (1:100, HCD56, BioLegend), CD16-PE/Cy7 (1:100, 3G8, BioLegend), NKG2D-FITC (1:100, 1D11, BioLegend), NKG2A-PE (1:100, 131411, R&D), CX3CR1-BV711 (1:100, 2A9-1, BioLegend).

Validation

To determine optimal staining and using optimal antibody concentrations, instrument gain settings were established for each parameter by evaluation of single-positive signals and confirmation that all positive events were below the maximum dynamic range of the instrument (7.2 log). Using established gains for all parameters, compensation was calculated, fluorescence minus one (FMO) controls, and single color controls were collected, to ensure downstream analysis accuracy of determined positive event populations. A minimum of 100,000 events were collected per sample to ensure robust breadth of population distributions.

# Plants

Seed stocks

n/a

Novel plant genotypes

n/a

Authentication

n/a

# Flow Cytometry

## Plots

Confirm that:

☒ The axis labels state the marker and fluorochrome used (e.g. CD4-FITC).

☒ The axis scales are clearly visible. Include numbers along axes only for bottom left plot of group (a 'group' is an analysis of identical markers).

☒ All plots are contour plots with outliers or pseudocolor plots.

☒ A numerical value for number of cells or percentage (with statistics) is provided.

## Methodology

Sample preparation

PBMC processing was adapted from Barcelo et al., 2018 44. Cryopreserved samples were submerged halfway for 60 seconds in a 37°C water bath. Immediately prior to full sample thaw 1 mL of pre-warmed (37°C) complete RPMI (RPMI, 10% FBS, 1% Pen/Strep) was added dropwise, pipetted against the tube wall. Thawed PBMCs were poured into a 15 mL conical tube containing 5 mL of pre-warmed (37°C) complete RMPI. Cryovials were rinsed with 2 mL of pre-warmed (37°C) complete RPMI and then poured into the previously used conical tube with cell mixture. PBMCs were incubated for 5 minutes in a 37°C water bath. PBMCs were then pelleted for 10 minutes at 1500 rpm, at room temperature. Supernatant was discarded and 1 mL of pre-warmed (37°C) complete RPMI with 50 U/mL of DNase (Roche, Cat# 04-716-728-001, 10 units/µL) was added, resuspension was done without pipetting. PBMCs were then incubated for 1 hour at 37°C in a water jacketed incubator (5% CO2, 95% humidity) with tube cap loosened. Following incubation PBMCs were pelleted and resuspended for counting in preparation for flow cytometry.

Instrument

NovoCyte Quanteon Flow Cytometer (Agilent Technologies) with 4 excitation lasers: 405 nm, 488 nm, 561 nm and 640 nm.

Software

Analysis of flow cytometry data was carried out using FlowJo 10.8 (BD Biosciences)

Cell population abundance

Prior to sample analysis, instrumentation performance was evaluated by the quality control (QC) procedure, experiments were only run if performance was optimal. To establish study standardization longitudinally, antibody titration was performed, and optimal antibody concentrations were determined. Using optimal antibody concentrations, instrument gain settings were established for each parameter by evaluation of single-positive signals and confirmation that all positive events were below the maximum dynamic range of the instrument (7.2 log). Using established gains for all parameters, compensation was calculated, fluorescence minus one (FMO) controls, and single color controls were collected, to ensure downstream analysis accuracy of determined positive event populations. A minimum of 100,000 events were collected per sample to ensure robust breadth of population distributions.

Gating strategy

Flow cytometry was employed using our 10-parameter panel. The gating strategy involved the establishment of population gating using quantitatively determined antibody titrations as depicted in the manuscript Figure 1A. Gates were set based on single color and fluorescence minus one (FMO) controls, with compensation to minimize fluorescent spill-over. To ensure accuracy, only live, single cell populations were analyzed to prevent non-specific binding artifacts or misrepresentation due to doublets, as described in the methods. The frequencies of cells expressing CD45+ (total leukocytes), CD3+CD14-CD19- (T

cells), CD3-CD14+CD19+ (B cells/monocytes), and CD3-CD14-CD19- (NK cells) were found to be comparable between PD and control groups (Figure 1B). To further validate the NK cell population, from CD3-CD14-CD19- gating, we identified three NK subsets with six NK subpopulations: CD56bright NK subset (CD56brightCD16- and CD56brightCD16dim NK subpopulations), CD56dim NK subset (CD56dimCD16bright, CD56dimCD16dim, and CD56dimCD16- NK subpopulations), and CD56- NK subset (CD56-CD16+ NK subpopulations)

☒ Tick this box to confirm that a figure exemplifying the gating strategy is provided in the Supplementary Information.
